# Human adipose tissue-derived multilineage progenitor cells exposed to oxidative stress induce neurite outgrowth in PC12 cells through p38 MAPK signaling

**DOI:** 10.1186/1471-2121-13-21

**Published:** 2012-08-07

**Authors:** Mariko Moriyama, Hiroyuki Moriyama, Ayaka Ueda, Yusuke Nishibata, Hanayuki Okura, Akihiro Ichinose, Akifumi Matsuyama, Takao Hayakawa

**Affiliations:** 1 Pharmaceutical Research and Technology Institute, Kinki University, 3-4-1 Kowakae, Higashi-Osaka, Osaka, 577-8502, Japan; 2 Department of Somatic Stem Cell Therapy and Health Policy, Foundation for Biomedical Research and Innovation, TRI305, 1-5-4 Minatojima-minamimachi, Chuo-ku, Kobe, Hyogo, 650-0047, Japan; 3 Department of Plastic Surgery, Kobe University Hospital, Kobe, Japan

**Keywords:** Human adipose-derived multilineage progenitor cells, Adult stem cells, Reactive oxygen species, p38 MAPK, Neurite outgrowth, BMP2, FGF2, Neurodegenerative disorders

## Abstract

**Background:**

Adipose tissues contain populations of pluripotent mesenchymal stem cells that also secrete various cytokines and growth factors to support repair of damaged tissues. In this study, we examined the role of oxidative stress on human adipose-derived multilineage progenitor cells (hADMPCs) in neurite outgrowth in cells of the rat pheochromocytoma cell line (PC12).

**Results:**

We found that glutathione depletion in hADMPCs, caused by treatment with buthionine sulfoximine (BSO), resulted in the promotion of neurite outgrowth in PC12 cells through upregulation of bone morphogenetic protein 2 (BMP2) and fibroblast growth factor 2 (FGF2) transcription in, and secretion from, hADMPCs. Addition of *N*-acetylcysteine, a precursor of the intracellular antioxidant glutathione, suppressed the BSO-mediated upregulation of BMP2 and FGF2. Moreover, BSO treatment caused phosphorylation of p38 MAPK in hADMPCs. Inhibition of p38 MAPK was sufficient to suppress BMP2 and FGF2 expression, while this expression was significantly upregulated by overexpression of a constitutively active form of MKK6, which is an upstream molecule from p38 MAPK.

**Conclusions:**

Our results clearly suggest that glutathione depletion, followed by accumulation of reactive oxygen species, stimulates the activation of p38 MAPK and subsequent expression of BMP2 and FGF2 in hADMPCs. Thus, transplantation of hADMPCs into neurodegenerative lesions such as stroke and Parkinson’s disease, in which the transplanted hADMPCs are exposed to oxidative stress, can be the basis for simple and safe therapies.

## Background

Mesenchymal stem cells (MSCs) are pluripotent stem cells that can differentiate into various types of cells [1-6]. These cells have been isolated from bone marrow [1], umbilical cord blood [2], and adipose tissue [3-6] and can be easily obtained and expanded *ex vivo* under appropriate culture conditions. Thus, MSCs are an attractive material for cell therapy and tissue engineering. Human adipose tissue-derived mesenchymal stem cells, also referred to as human adipose tissue-derived multilineage progenitor cells (hADMPCs), are especially advantageous because they can be easily and safely obtained from lipoaspirates, and the ethical issues surrounding other sources of stem cells can be avoided [4-6]. Moreover, hADMPCs have more pluripotent properties for regenerative medical applications than other stem cells, since these cells have been reported to have the ability to migrate to the injured area and differentiate into hepatocytes [4], cardiomyoblasts [5], pancreatic cells [7], and neuronal cells [8-10]. In addition, it is known that hADMPCs secrete a wide variety of cytokines and growth factors necessary for tissue regeneration including nerve growth factor (NGF), brain-derived neurotrophic factor (BDNF), fibroblast growth factors (FGFs), vascular endothelial growth factor (VEGF) and hepatocyte growth factor (HGF) [11-14].

Recently, several groups have reported that hADMPCs facilitate neurological recovery in experimental models of stroke [9,10,15] and Parkinson’s disease [16]. Despite the superiority of hADMPCs over other stem cells, the potential use of hADMPCs for the treatment of these neurodegenerative disorders has not been fully investigated. It has been reported that administration of hADMPCs in animal models of acute ischemic stroke markedly decreased brain infarct size, improved neurological function by enhancing angiogenesis and neurogenesis, and showed anti-inflammatory and anti-apoptotic effects [9,10]. These effects were due in part to increased secretion levels of VEGF, HGF and bFGF under hypoxic conditions [13], indicating the role of hADMPCs in reducing the severity of hypoxia-ischemic lesions.

In addition to hypoxic stress, ischemic lesions are generally subject to inflammation, which leads to the generation of reactive oxygen species (ROS) [17,18]. ROS are generated as a natural byproduct of normal aerobic metabolism, and mitochondrial respiration, together with oxidative enzymes such as plasma membrane oxidase, is considered to be the major intracellular source of ROS production [19]. Although appropriate levels of ROS play an important role in several physiological processes, oxidative damage initiated by excessive ROS causes many pathological conditions including inflammation, atherosclerosis, aging, and cancer. Neuronal cells are especially vulnerable to oxidative stress, and numerous studies have examined the crucial roles of oxidative stress in neurodegenerative disorders such as stroke [17,18], Alzheimer’s disease [20,21], and Parkinson’s disease [22,23]. In these diseases, microglia, the macrophages of the central nervous system (CNS), are activated in response to a local inflammation [24] and generate large amounts of reactive oxygen and nitrogen species, thereby exposing nearby neurons to stress [18,25]. Thus, the influence of oxidative stress generated by neurodegenerative lesion on hADMPCs needs to be further studied.

In this study, we examined the role of oxidative stress on hADMPCs in neurite outgrowth in cells of the rat pheochromocytoma cell line (PC12). Upon treatment with buthionine sulfoximine (BSO), an inhibitor of the rate-limiting enzyme in the synthesis of glutathione, hADMPCs accumulated ROS, which resulted in the upregulation of expression levels of the neurotrophic factors BMP2 and FGF2. Our present data thus provide new insights into understanding the mechanism of how hADMPCs exposed to oxidative stress contribute to neurogenesis, and this may explain the effects of stem cell transplantation therapy with hADMPCs in treating ischemic stroke.

## Results

### hADMPCs exposed to oxidative stress stimulate neurite outgrowth in PC12 cells

**Figure 1:**
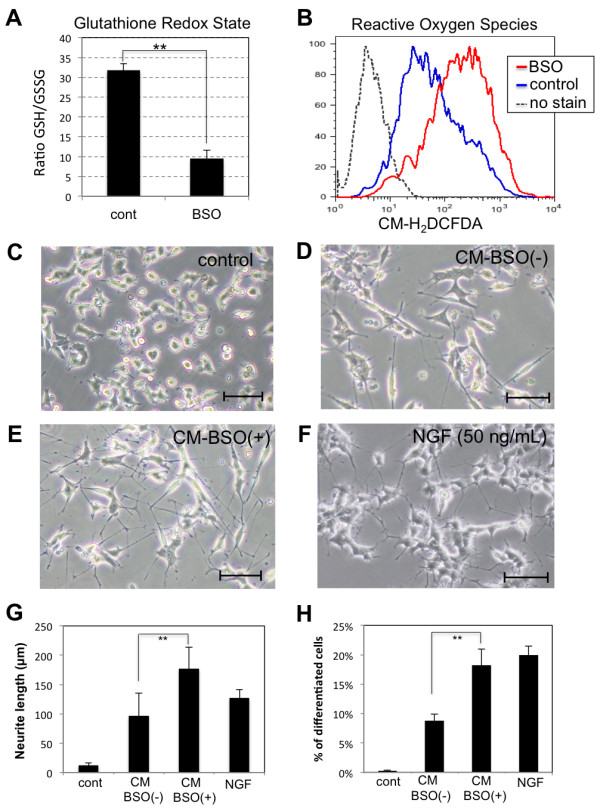
** Conditioned medium from hADMPCs exposed to oxidative stress induces neurite outgrowth in PC12 cells. (A, B)** Decrease of the reduced/oxidized glutathione ratios and increase in the intracellular ROS levels in hADMPCs treated with BSO. hADMPCs were treated with 1 mM BSO for 16 h, and cellular GSH/GSSG levels (**A**) or ROS (H_2_O_2_) levels (**B**) were analyzed. **(C-G)** Induction of neurite outgrowth in PC12 cells by conditioned medium from BSO-treated hADMPCs. PC12 cells were induced to differentiation by changing medium to differentiation medium alone (**C**), CM-BSO (−) (**D**), CM-BSO (+) (**E**), or differentiation medium with NGF (50 ng/mL) (**F**) for 2 days. Scale bars, 200 μm. (**G**) One hundred individual neurites were measured in each sample using Dynamic Cell Count Analyzer BZ-H1C (Keyence, Osaka, Japan) and average neurite length was calculated. **, P < 0.01 (Student’s t test). **(H)** Percentage of neurite-bearing PC12 cells. A cell was scored positive for bearing neurites if it has a thin neurite extension that is double the length of the cell body diameter. A total of 500–600 cells in each sample were counted. **, P < 0.01 (Student’s t test).

hADMPCs were treated with 1 mM BSO for 24 h; a group of hADMPCs that were not given any treatment was used as the control group. As shown in Figure 1A and B, BSO treatment resulted in significant reduction of intracellular reduced glutathione levels, followed by accumulation of intracellular reactive oxygen species (ROS) in hADMPCs. To investigate whether accumulation of ROS affects secretion of cytokines from hADMPCs, conditioned medium from BSO-treated (CM-BSO (+)) or BSO-untreated (CM-BSO (−)) hADMPCs was added to PC12 cells. As expected, addition of NGF significantly induced neurite outgrowth in the PC12 cells (Figure 1F, G, H). hADMPCs, like other mesenchymal stem cells derived from bone marrow or adipose tissue, may secrete many cytokines including NGF, BDNF and FGF2, and this may account for the slight induction of neurite outgrowth seen in the CM-BSO (−) treated cells (Figure 1D, G, H). In contrast, the number and length of neurite outgrowth of PC12 cells in CM-BSO (+) (Figure 1E) was markedly enhanced compared with those in CM-BSO (−) (Figure 1D, E, G, H).

### Conditioned medium from BSO-treated hADMPCs activates Erk1/2 MAPK and Smad signaling in PC12 cells

**Figure 2:**
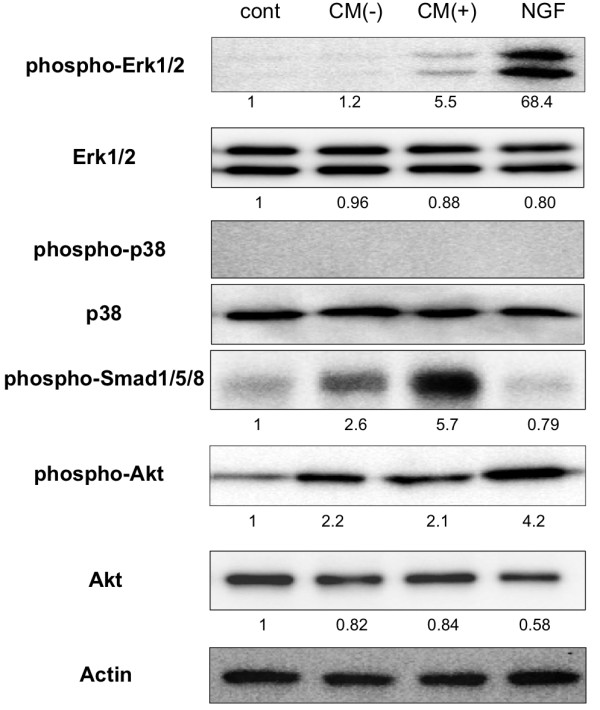
** Erk1/2 MAPK and Smad1/5/8 are activated in PC12 cells cultured in conditioned medium from BSO-treated hADMPCs.** Western blot analysis of PC12 cells cultured in differentiation medium alone (cont), CM-BSO (−), CM-BSO (+), or differentiation medium with NGF (50 ng/mL) for 1 h. Proteins extracted from each cell culture were resolved by SDS-PAGE, transferred to a membrane, and probed with anti-phosphorylated Erk1/2 (phospho Erk1/2), anti-Erk1/2, anti-phosphorylated p38 (phospho p38), anti-p38, anti-phosphorylated Smad1/5/8 (phospho Smad1/5/8), anti-phosphorylated Akt (phospho Ark) and anti-Akt. Actin was analyzed as an internal control. Numbers below blots indicate relative band intensities as determined by the ImageJ software.

To investigate which intracellular signaling pathways were involved in the neurite outgrowth of PC12 cells in CM-BSO (+), we used western blotting to determine the phosphorylation levels of Erk1/2 MAPK, p38 MAPK, Smad1/5/8 and Akt in PC12 cells in various culture conditions. NGF significantly activated Erk1/2 MAPK and Akt signaling pathway (Figure 2). In contrast, Erk1/2 MAPK was not activated in PC12 cells exposed to CM-BSO (−), while an increase in phosphorylated Smad1/5/8 was observed. Interestingly, CM-BSO (+) treatment led to both a significant increase in Smad1/5/8 phosphorylation levels as well as activation of the Erk1/2 MAPK signaling pathway in PC12 cells (Figure 2). Akt was 2-fold activated in both CM-BSO (−) and CM-BSO (+) treated PC12 cells, but no significant difference between the 2 groups was observed.

### FGF2 and BMP2 are upregulated through p38 MAPK signaling in hADMPCs exposed to oxidative stress

**Figure 3:**
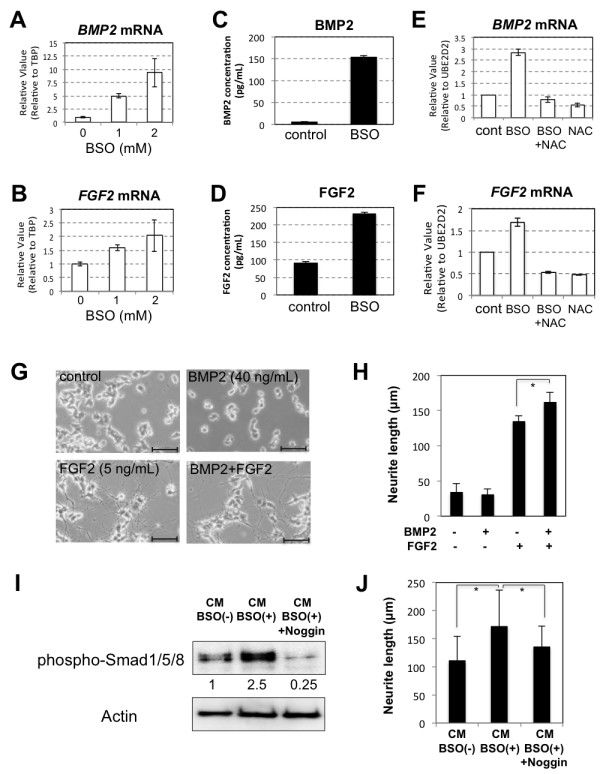
** Transcription and secretion of BMP2 and FGF2 were increased in hADMPCs exposed to oxidative stress. (A, B)** Upregulation of *BMP2* (**A**) and *FGF2* (**B**) mRNA in hADMPCs by BSO in a dose-dependent manner. **(C, D)** Secretion of BMP2 (**C**) and FGF2 (**D**) from hADMPCs in medium alone (cont) or with addition of 1 mM BSO (BSO) was analyzed by ELISA. **(E, F)** NAC treatment repressed the expression levels of *BMP2* and *FGF2* upregulated by BSO to the control levels. Expression of *BMP2* (E) and *FGF2* (F) mRNA was analyzed by q-PCR. cDNA was generated from total RNA extracted from hADMPCs (cont), hADMPCs treated with 1 mM BSO (BSO), 1 mM BSO + 5 mM NAC (BSO + NAC), and 5 mM NAC (NAC). The most reliable internal control gene was determined using the geNorm Software. **(G, H)** PC12 cells were cultured in differentiation medium alone (control), or differentiation medium supplemented with BMP2 (40 ng/mL), FGF2 (5 ng/mL), or both BMP2 and FGF2 (BMP2 + FGF2) for 2 days. (**G**) Representative images of neurite outgrowth in PC12 cells. Scale bars, 200 μm. (**H**) One hundred individual neurites were measured in each sample using Dynamic Cell Count Analyzer BZ-H1C (Keyence) and average neurite length was calculated. *, P < 0.05 (Student’s t test). **(I, J)** PC12 cells were cultured in CM-BSO (−), CM-BSO (+), or CM-BSO (+) added with recombinant murine Noggin (200 ng/mL). **(I)** Western blot analysis of PC12 cells 1 h after CM treatment. Proteins extracted from each sample were resolved by SDS-PAGE, transferred to a membrane, and probed with anti-phosphorylated Smad1/5/8 (phospho-Smad1/5/8) and anti-Actin. Numbers below blots indicate relative band intensities as determined by the ImageJ software. (**J**) Two days after CM treatment, 100 individual neurites in PC12 cells were measured in each sample using Dynamic Cell Count Analyzer BZ-H1C (Keyence) and average neurite length was calculated. *, P < 0.05 (Student’s t test).

We next examined which growth factors or cytokines from BSO-treated hADMPCs were involved in stimulation of neurite outgrowth. We found that both mRNA (Figure 3A and B) and protein (Figure 3C and D) levels for BMP2 and FGF2 were markedly increased in hADMPCs treated with BSO. To determine if this upregulation was caused by ROS, all cells were exposed to the antioxidant *N*-acetylcysteine (NAC). As we expected, addition of NAC to BSO-treated hADMPCs reduced the expression levels of BMP2 and FGF2 to control levels (Figure 3E and F). As BMP2 together with FGF2 has previously been shown to induce neurite outgrowth in PC12 cells [26,27], we examined the effect of BMP2 and FGF2 on neurite outgrowth. We confirmed that PC12 cells did not differentiate effectively by BMP2 treatment alone, but BMP2 significantly augmented FGF2-induced neurite outgrowth in PC12 cells (Figure 3G and H), as previously reported. Moreover, in order to confirm the effect of BMP2 on neurite outgrowth in PC12 cells, 200 ng/mL of Noggin, an antagonist of BMP signaling, was added to CM-BSO(+). Addition of Noggin significantly suppressed the CM-BSO (+)-evoked phosphorylation of Smad1/5/8 (Figure 3I) and shortened the length of neurite outgrowth in PC12 cells (Figure 3J). 

**Figure 4:**
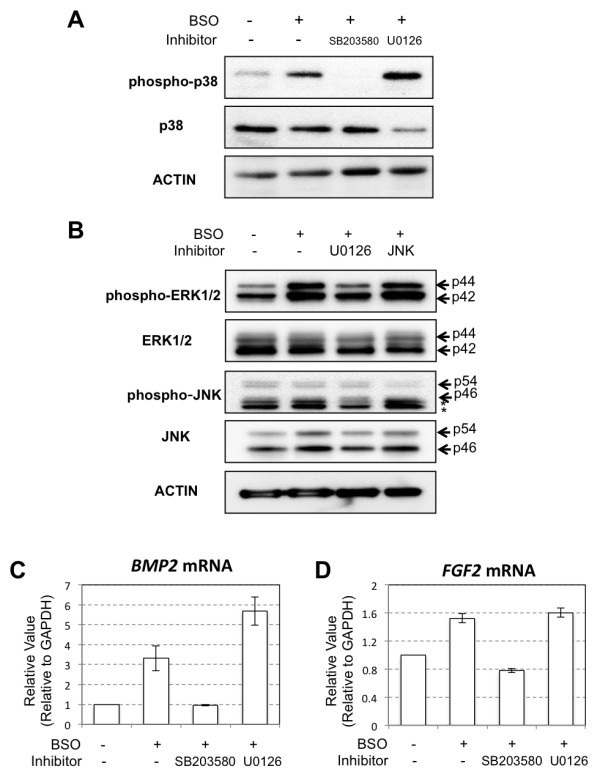
** BMP2 and FGF2 were upregulated through activation of p38 MAPK.** Inhibiton of p38 MAPK resulted in the supression of *BMP2* and *FGF2* transcripts upregulated by BSO treatment in hADMPCs. hADMPCs were pre-treated with 10 μM of SB203580, 10 μM of U0126 or 10 μM of JNK inhibitor II for 2 h followed by 1 mM BSO treatment for 16 h. The medium was replaced with fresh culture medium and the cells were cultured for another 2 days. **(A)** Western blot analysis of p38 MAPK activation in hADMPCs. **(B)** Western blot analysis of ERK1/2 MAPK, JNK SAPK activation in hADMPCs. **(C, D)** Transcription levels of *BMP2* (**C**) and *FGF2* (**D**) were analyzed by q-PCR. The most reliable internal control gene was determined using the geNorm Software.

To address the question of which intracellular signaling pathways are affected by oxidative stress in hADMPCs, we focused on MAPK signaling since previous studies had suggested that accumulation of ROS in cells led to the activation of Erk1/2, p38, and JNK MAPK [28,29]. Western blotting revealed that BSO treatment markedly activated the p38 MAPK pathway; SB203580 could inhibit the activation, and U0126 treatment stimulated the activation (Figure 4A). ERK1/2 MAPK was significantly phosphorylated by BSO treatment, and ERK1/2 activation was reduced to the control level by treatment with U0126 (Figure 4B). In contrast, JNK activation was not observed in BSO-treated hADMPCs (Figure 4B). Therefore, we further investigated the relationship between increases in BMP2 and FGF2 expression and activation of the p38 and ERK1/2 MAPK signaling pathways by oxidative stress. Treatment with the p38 MAPK inhibitor SB203580 dramatically downregulated the expression levels of *BMP2* and *FGF2* to control levels (Figure 4C and D). In contrast, the Erk1/2 MAPK inhibitor U0126 had no effect on *FGF2* expression levels and led to a slight increase in *BMP2* expression (Figure 4C and D). 

### MKK6-mediated activation of p38 MAPK increases BMP2 and FGF2 expression in hADMPCs

**Figure 5:**
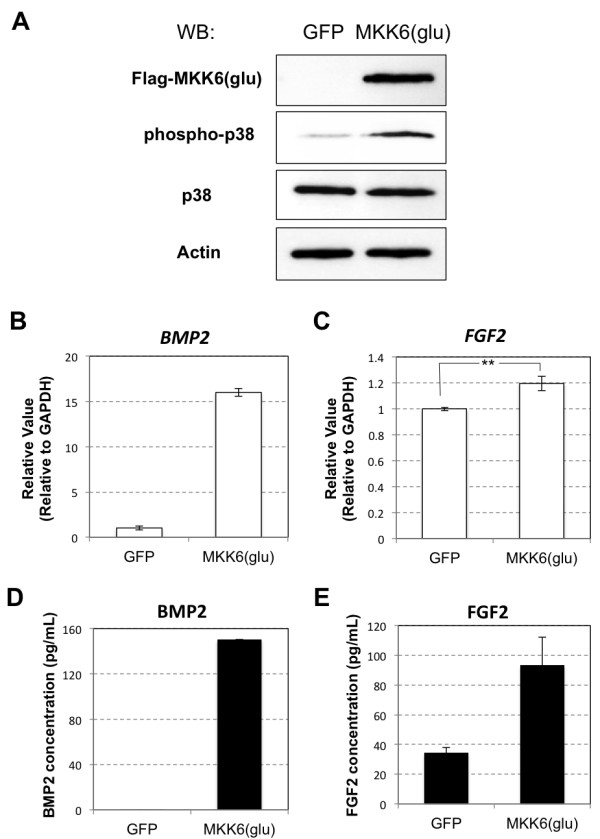
** Activation of p38 MAPK by a constitutively active form of MKK6 resulted in elevated expression of BMP2 and FGF2.(A)** A lentiviral vector expressing Flag-tagged MKK6 (glu) was transfected into hADMPCs. Expression of Flag-tagged MKK6 (glu), phosphorylated p38 MAPK and p38 MAPK was analyzed by western blotting. A CSII-EF-EGFP lentiviral vector was infected as a control (GFP). Actin was detected as an internal control. **(B, C)** Transcriptional levels of *BMP2* (**B**) and *FGF2* (**C**) were analyzed by q-PCR. The most reliable internal control gene was determined using the geNorm Software. **(D, E)** BMP2 (**D**) and FGF2 (**E**) secretion was analyzed by ELISA.

To further confirm the involvement of p38 MAPK in the regulation of BMP2 and FGF2, hADMPCs were transduced with a lentiviral vector expressing constitutively active MKK6 (MKK6 (glu)) [30] from an EF1α promoter. As shown in Figure 5A, lentiviral transduction of MKK6 (glu) led to expression of Flag-tagged MKK6 (glu) in hADMPCs. Moreover, the expression of MKK6 (glu) resulted in activation of p38 MAPK as expected [30] (Figure 5A), and upregulation of BMP2 and FGF2 expression (Figure 5B-E). 

### NF-κB is not activated in hADMPCs exposed to oxidative stress

**Figure 6:**
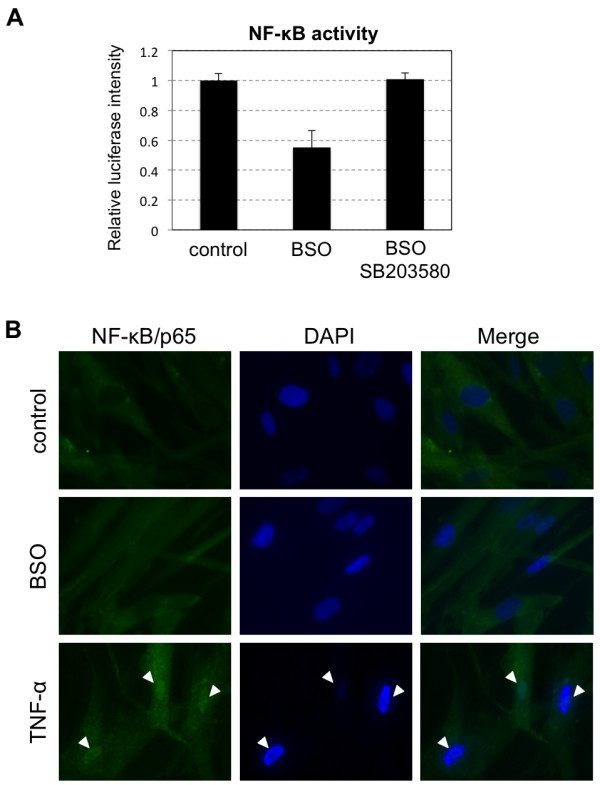
** Transcriptional activity of NF-κB was not stimulated by oxidative stress.** hADMPCs were transfected with pNF-κB-Luc and were treated with 10 μM of SB203580 or 0.1% DMSO for 2 h followed by addition of 1 mM of BSO. After 24 h, the medium was changed to fresh medium and cells were cultured for an additional 2 days. **(A)** The activity of NF-κB was measured by the intensity of luciferase activity driven from a minimal promoter containing tandem repeats of the NFκB transcriptional response element. Data shown represent the average of 3 independent experiments. **(B)** Immunocytochemical analysis against NF-κB/p65 (green). Blue staining represents nuclear staining by DAPI. Note that nuclear localization of NF-κB/p65 (white arrowhead) is only observed in hADMPCs treated with 20 ng/mL of TNF-α.

It has been reported that NF-κB directly binds to the *BMP2* promoter to induce its expression [31], and MSK1, a downstream molecule of p38 MAPK, is involved in NF-κB transactivation [32]. Therefore, we hypothesized that p38 MAPK-mediated activation of NF-κB might contribute to elevated expression of *BMP2* mRNA. To confirm this hypothesis, transcriptional activation of NF-κB was examined by measuring luciferase activity driven by the synthetic NF-κB response element. We found that transcriptional activity of NF-κB was not stimulated by BSO treatment (Figure 6A), and immunocytochemical analysis also revealed that NF-κB was not activated (nuclear localization of NF-κB/p65 was rarely observed) in BSO-treated hADMPCs (Figure 6B). These results suggested that elevated expression of *BMP2* mRNA is not mediated by NF-κB signaling. 

**Figure 7:**
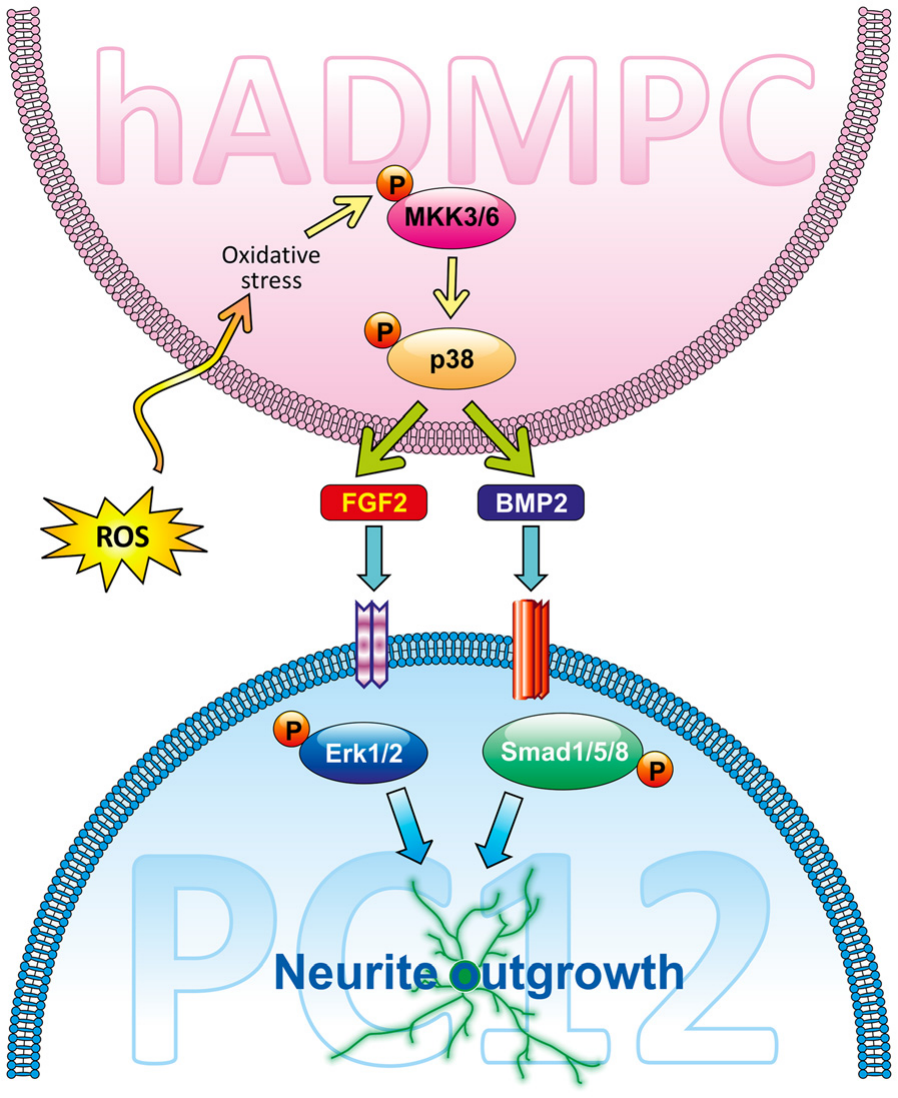
** Model of this study.** A schematic illustration of the results of this study is shown. Oxidative stress by BSO treatment in hADMPCs results in p38 MAPK activation, which then leads to BMP2 and FGF2 expression and secretion. The secreted factors then bind to the receptors on PC12 cells, facilitating neural differentiation in these cells.

Our current data thus demonstrate the crucial role of ROS, via activation of the p38 MAPK signaling pathway, in regulating expression levels of the neurotrophic factors BMP2 and FGF2 in hADMPCs. The overall model that we propose, based upon our findings, is shown in Figure 7.

## Discussion

In this study, we investigated the effect of oxidative stress in hADMPCs on the induction of neuronal differentiation. Such mechanisms may explain how administration of hADMPCs to neurodegenerative lesions enhances endogenous repair mechanisms via neurogenesis of endogenous neural progenitor and stem cells. Damaged tissues, such as the brain tissue of patients who have suffered from ischemic stroke, are subject to inflammation and the generation of reactive oxygen species (ROS) [17,18]. Our data demonstrated that hADMPCs, when exposed to oxidative stress, facilitate neuronal differentiation in rat pheochromocytoma cell line PC12 cells by upregulation of fibroblast growth factor 2 (FGF2) and bone morphogenetic protein 2 (BMP2) secretion through p38 MAPK activation.

Our results show that BMP2 and FGF2 were upregulated in hADMPCs when exposed to buthionine sulfoximine (BSO), a glutathione-synthesis inhibitor that leads to oxidative stress. These findings may have therapeutic implications in neurodegenerative diseases. We concluded that BMP2 and FGF2 secreted from hADMPCs that had been exposed to oxidative stress were the main inducers of neurite outgrowth in PC12 cells. Erk1/2 and Smad1/5/8 were significantly activated in these cells (Figure 2), while other growth factors known to induce neurite outgrowth in PC12 cells such as nerve growth factor (NGF) and vascular endothelial growth factor (VEGF) were not observed to be upregulated by BSO treatment (data not shown). We confirmed that BMP2 enhanced the effect that FGF2 had on the differentiation of PC12 cells (Figure 3), supporting our idea that hADMPCs under oxidative stress conditions secrete BMP2 and FGF2 and that this contributes to neuronal differentiation. Consistent with our conclusions, it has been reported that BMP2, via activation of a Smad signaling pathway, facilitated FGF2-induced neuronal differentiation in PC12 cells [26,27]. However, since hADMPCs have been reported to secrete many growth factor including NGF, VEGF, HGF, and IGF [11,15,33], we cannot exclude the possibility that BMP2 and FGF2 are acting cooperatively with these growth factors to facilitate neurite outgrowth in PC12 cells. Thus, the precise molecular mechanisms of induction of PC12 differentiation and the precise expression profiles in BSO-treated hADMPCs need to be further investigated.

Recently, BMP signaling through Smad1/5/8 has been reported to contribute to neurite outgrowth in dorsal root ganglion neurons both in vitro and in vivo [34,35]. Moreover, BMP2 has been shown to have neurotrophic effects on midbrain dopaminergic neurons [36], ventral mesencephalic neurons [37], mouse embryonic striatal neurons [38], and nitrergic and catecholaminergic enteric neurons [39]. Moreover, FGF2 is trophic for neurons, glias, and endothelial cells in the central nervous system. FGF2 also prevents downregulation of the anti-apoptotic protein Bcl-2 in ischemic brain tissue and limits excitotoxic damage to the brain through an activin-dependent mechanism [40]. These findings are consistent with our hypothesis that hADMPCs secret BMP2 and FGF2 to induce neurogenesis in neurodegenerative lesions in response to oxidative stress.

As it has been shown that ROS activate ERKs, JNKs, and p38 MAPKs [28,29], we examined the MAPK signaling pathway in hADMPCs exposed to oxidative stress and found that BSO treatment resulted in significant activation of ERK1/2 and p38 MAPK. Intriguingly, addition of SB203580, a specific inhibitor of p38 MAPK, but not the ERK inhibitor U0126, suppressed *BMP2* and *FGF2* expression in BSO-treated hADMPCs to control levels (Figure 4), suggesting that p38 MAPK was contributing to upregulation of BMP2 and FGF2 in hADMPCs when exposed to oxidative stress. Moreover, lentiviral transduction of the constitutively active form of MKK6, a MAPKK that selectively activates p38 MAPK isoforms [30], resulted in upregulation of BMP2 and FGF2 and this also demonstrated the crucial role of the p38 MAPK cascade in the regulation of BMP2 and FGF2. In primary human endothelial cells, p38-dependent regulation of BMP2 expression was reported previously. Viemann *et al.*[41] investigated the genes that were induced by inflammatory stimulation with tumor necrosis factor α (TNF-α) and classified these genes into 2 categories based on whether they were regulated in an NF-κB-dependent or p38 MAPK-dependent manner. Consistent with our findings, they found that significant induction of BMP2 expression by TNF-α was markedly suppressed by SB202190, an inhibitor of p38 MAPK. These results support the hypothesis that activation of the p38 MAPK pathway in hADMPCs in response to inflammation surrounding neurodegenerative lesions leads to induction of BMP2 and FGF2, which in turn support regeneration of neuronal cells.

It has been known that NF-κB directly binds to the BMP2 promoter to induce its expression [31], and MSK1, a downstream molecule of p38 MAPK, is involved in NF-κB transactivation [32]. However, we did not observe an elevation of NF-κB transcriptional activity in hADMPCs when they were exposed to oxidative stress (Figure 6). The mechanism of p38-dependent regulation of gene expression is not completely understood, and the precise mechanism by which p38 MAPK regulates the expression of BMP2 and FGF2 remains to be determined.

In this study, we also found that suppression of ERK1/2 MAPK by U0126 in BSO-treated hADMPCs resulted in slight activation of p38 MAPK (Figure 4A). Consistent with this, the expression level of *BMP2* mRNA was also upregulated when cells exposed to oxidative stress were pretreated with U0126 (Figure 4C). Previously, “seesaw cross-talk” between ERK and p38 MAPK signaling has been reported; i.e., the MEK inhibitor caused a decrease in the phosphorylation level of ERK and an increase in that of p38, whereas the p38 inhibitor had the opposite effect [42-44]. We did not investigate the phosphorylation of ERK1/2 in SB203580-treated hADMPCs, but it may be possible that seesaw cross-talk also occurs in our system.

## Conclusions

In summary, the results obtained in this study have demonstrated the potential use of hADMPCs for the treatment of neurodegenerative diseases such as ischemic stroke, Parkinson’s disease, Alzheimer’s disease, and spinal cord injury, in which the transplanted hADMPCs might be exposed to oxidative stress. Moreover, the p38-dependent modulation of BMP2 and FGF2 expression observed in this study is expected to be a new therapeutic target for neurodegenerative disorders.

## Materials and methods

### Adipose tissue samples

Subcutaneous adipose tissue samples (10–50 g, each) were resected during plastic surgery in 5 females (age, 20–60 years) as excess discards. The study protocol was approved by the Review Board for Human Research of Kobe University Graduate School of Medicine, Foundation for Biomedical Research and Innovation and Kinki University Pharmaceutical Research and Technology Institute (reference number: 10–005). Each subject provided a signed informed consent.

### Cell culture

PC12 cells were obtained from the Health Science Research Resources Bank (Osaka, Japan) and maintained in RPMI1640 media supplemented with 10% heat-inactivated horse serum and 5% fetal bovine serum. For differentiation, the cells were plated in 6-well culture plates coated with collagen type I (Nitta Gelatin, Osaka, Japan) and the medium was replaced with differentiation medium (RPMI1640 supplemented with 1% horse serum and 0.5% fetal bovine serum) or conditioned medium from hADMPCs. NGF (50 ng/mL), BMP2 (40 ng/mL) or FGF2 (5 ng/mL) were added to the differentiation medium. Recombinant murine Noggin (200 ng/mL: PeproTech, NJ, USA) was added to conditioned medium from BSO-treated hADMPCs. hADMPCs were isolated as previously reported [4-6,45,46] and maintained in a medium containing 60% DMEM-low glucose, 40% MCDB-201 medium (Sigma Aldrich, St. Louis, MO, USA), 1× insulin-transferrin-selenium (Gibco Invitrogen, NY, USA), 1 nM dexamethasone (Sigma Aldrich), 100 mM ascorbic acid 2-phosphate (Wako, Osaka, Japan), 10 ng/mL epidermal growth factor (PeproTech), and 5% fetal bovine serum. The cells were plated to a density of 5 × 10^3^ cells/cm^2^ on fibronectin-coated dishes, and the medium was replaced every 2 days.

### Preparation of conditioned medium from hADMPCs

Two days after plating, hADMPCs were treated with BSO (concentrations used were varied in each experiment and are indicated in the results and figure legends) for 16 h. The medium was replaced with fresh culture medium for 2 days followed by replacement with PC12 cell differentiation medium. After 2 more days, the medium was removed for use as conditioned medium. For preparation of the conditioned medium from hADMPCs in which one of the three, p38, Erk1/2, or JNK MAPK, was inhibited, hADMPCs were pretreated with 10 μM SB203580 (Promega, WI, USA), 10 μM U0126 (Promega), or 10 μM JNK inhibitor II (EMD4 Bioscience, CA, USA), respectively, for 2 h and subsequently treated with 1 mM BSO.

### Measurement of GSH/GSSG ratio

Ratios of reduced glutathione (GSH) to oxidized glutathione (GSSG) were measured using the GSH/GSSG-Glo assay kit (Promega) following the manufacturer’s protocol.

### Measurement of reactive oxygen species production

Cells were harvested and incubated with 10 μM 5-(and-6)-chloromethyl-2′,7′-dichlorodihydrofluorescein diacetate, acetyl ester (CM-H_2_DCFDA). The amount of intracellular ROS production was proportional to green fluorescence, as analyzed with a Guava easyCyte 8HT flow cytometer (Millipore) using an argon laser at 488 nm and a 525/30 nm band pass filter, and dead cells were excluded with the LIVE/DEAD fixable far red dead cell stain kit (Invitrogen).

### Western blot analysis

Cells were washed with ice-cold phosphate-buffered saline and lysed with M-PER Mammalian Protein Extraction Reagent (Thermo Scientific Pierce, IL, USA) following the manufacturer’s instructions. Equal amounts of proteins were separated by sodium dodecylsulfate polyacrylamide gel electrophoresis (SDS-PAGE), transferred to polyvinylidene fluoride (PVDF) membranes (Immobilon-P; Millipore, MA, USA), and probed with antibodies against phospho-Erk1/2 (#4370), Erk1/2 (#4695), phospho-38 (#9215), p38 (#9212), phospho-Smad1/5/8 (#9511), phospho-Akt (#4060), Akt (#4691), phospho-JNK (#9251), JNK (#9258) (all from Cell Signaling Technology, MA, USA) and actin (Millipore). Horseradish peroxidase (HRP)-conjugated anti-rabbit and anti-mouse secondary antibodies (Cell Signaling Technology, Danvers, MA, USA) were used as probes and immunoreactive bands were visualized with the Immobilon Western Chemiluminescent HRP substrate (Millipore). The band intensity was measured using ImageJ software.

### RNA extraction, cDNA generation, and quantitative polymerase chain reaction (q-PCR)

Total RNA was extracted using the RNeasy Mini Kit (Qiagen, Hilden, Germany) following the manufacturer’s instructions. cDNA was generated from 1 μg of total RNA using the Verso cDNA Synthesis Kit (Thermo Scientific) and purified with the MinElute PCR Purification Kit (Qiagen). Q-PCR analysis was carried out using the SsoFast EvaGreen supermix (Bio-Rad, CA, USA) according to the manufacturer’s protocols. The relative expression value of each gene was calculated using a ΔΔCt method and the most reliable internal control gene was determined using the geNorm Software (http://medgen.ugent.be/~jvdesomp/genorm/). Details of the primers used in these experiments are available on request.

### Enzyme-linked immunosorbent assay

Enzyme-linked immunosorbent assay (ELISA) was performed using the Quantikine BMP-2 Immunoassay System and Quantikine FGF-2 Immunoassay System (R&D Systems, MN, USA) following the manufacturer’s protocols.

### Plasmid construction and lentivirus production

Flag-tagged MKK6 (glu) [30] was provided by Addgene (pcDNA3-Flag MKK6 (glu); Addgene plasmid 13518). Flag-tagged MKK6 (glu) was cloned into a pENTR11 vector (Invitrogen). An iresGFP fragment was subsequently cloned into the plasmid to produce the entry vector pENTR11-MKK6 (glu)-iresGFP. The entry vector and CSII-EF-RfA (kindly provided by Dr. Miyoshi, RIKEN BioResource Center, Tsukuba, Japan) were incubated with LR clonase II enzyme mix (Invitrogen) to generate CSII-EF-MKK6 (glu)-iresGFP. The resultant plasmid was mixed with packaging plasmids (pCAG-HIVg/p and pCMV-VSVG-RSV-Rev, kindly provided by Dr. Miyoshi) and transfected into 293 T cells. The supernatant medium, which contained lentiviral vectors, was collected 2 days after transduction and concentrated by centrifugation (6000 G, 15 h, 4°C).

### Luciferase assay

hADMPCs were transfected with pGL4.74 (Promega) and either pTAL-Luc or pNF-κB-Luc by TransIT-2020 (TaKaRa-Bio). The cells were then treated with 10 μM of SB203580 or 0.1% DMSO for 2 h followed by addition of 1 mM of BSO. After 24 h, the medium was changed to fresh medium and cells were cultured for an additional 2 days. The activity of NF-κB was measured using the Dual Luciferases Assay System (Promega) according to the manufacturer’s protocol.

### Immunocytochemistry

hADMPCs were fixed with 4% paraformaldehyde in PBS for 10 min at 4°C and then washed 3 times in PBS. Blocking was performed with PBSMT (PBS containing 0.1% Triton X-100, 2% Skim Milk) for 1 h at room temperature. The cells were then incubated with rabbit monoclonal antibody against NF-κB p65 (Cell Signaling; #8242; 1/100 dilution) overnight at 4°C. After washing with PBS, cells were incubated with Alexa 488 conjugated anti-rabbit IgG (Invitrogen; 1/1000 dilution) for 1 h. The cells were counterstained with 4′-6-diamidino-2-phenylindole (DAPI) (Invitrogen) to identify cellular nuclei.

## Competing interests

None of the authors have any competing interests related to the manuscript.

## Authors’ contributions

MM carried out the FACS analysis, qPCR analysis, ELISA, immunofluorescent staining, and cell culture, participated in the study design, and drafted the manuscript. HM participated in the study design, carried out the western blot analysis, luciferase assay, and cell culture, and drafted the manuscript. AU carried out western blot analysis, constructed the plasmids, and generated the lentiviral vectors. YN carried out qPCR analysis and performed the statistical analysis. AI resected subcutaneous adipose tissue samples during plastic surgery. HO and AM isolated hADMPCs from human adipose tissues. TH conceived the study, participated in its design and coordination, and helped to draft the manuscript. All authors read and approved the final manuscript.
